# Endoscopic Ultrasound-Guided Anastomoses of the Gastrointestinal Tract: A Multicentric Experience

**DOI:** 10.3390/cancers17050910

**Published:** 2025-03-06

**Authors:** Giacomo Emanuele Maria Rizzo, Chiara Coluccio, Edoardo Forti, Alessandro Fugazza, Cecilia Binda, Giuseppe Vanella, Francesco Maria Di Matteo, Stefano Francesco Crinò, Andrea Lisotti, Marcello Fabio Maida, Giovanni Aragona, Aurelio Mauro, Alessandro Repici, Andrea Anderloni, Carlo Fabbri, Ilaria Tarantino, on behalf of the I-EUS Group

**Affiliations:** 1 Gastroenterology and Endoscopy Unit, Istituto Mediterraneo per i Trapianti e Terapie di alta Specializzazione—IRCCS ISMETT, 90127 Palermo, Italy; itarantino@ismett.edu; 2 Department of Precision Medicine in Medical, Surgical and Critical Care (Me.Pre.C.C.), University of Palermo, 90127 Palermo, Italy; 3 Gastroenterology and Digestive Endoscopy Unit, Forlì-Cesena Hospitals, AUSL Romagna, 47121 Ravenna, Italy; 4 Digestive and Operative Endoscopy Unit, ASST Grande Ospedale Metropolitano Niguarda, 20162 Milan, Italy; 5 Department of Biomedical Sciences, Humanitas University, Via Rita Levi Montalcini 4, Pieve Emanuele, 20090 Milan, Italy; 6 Endoscopy Unit, IRCCS Humanitas Research Hospital, Rozzano, 20089 Milan, Italy; 7 Pancreatobiliary Endoscopy and Endosonography Division, Pancreas Translational and Clinical Research Centre, IRCCS San Raffaele Scientific Institute, 20132 Milan, Italy; 8 Operative Endoscopy Department, Campus Bio-Medico University Hospital, 00128 Rome, Italy; 9 Gastroenterology and Digestive Endoscopy Unit, The Pancreas Institute, 37134 Verona, Italy; 10 Gastroenterology Unit, Hospital of Imola, University of Bologna, 40026 Imola, Italy; 11 Department of Medicine and Surgery, University of Enna ‘Kore’, 94100 Enna, Italy; 12 Gastroenterology and Hepatology Unit, Ospedale Civile, AUSL Piacenza, 29121 Piacenza, Italy; 13 Gastroenterology and Digestive Endoscopy Unit Fondazione I.R.C.C.S. Policlinico San Matteo, 27100 Pavia, Italy; 14 Department of Internal Medicine and Medical Therapeutics, University of Pavia, 27100 Pavia, Italy

**Keywords:** afferent limb syndrome, endoscopic ultrasound, endoscopy, gastric outlet obstruction, gastroenteroanastomosis

## Abstract

This multicenter retrospective study included patients undergoing EUS-guided GI anastomoses from 2016 to 2023. Indications for EUS-guided anastomosis were GOO, ALS or patients with altered anatomy needing endoscopic interventions. The primary outcome was technical success, while secondary outcomes included clinical success, safety, lumen-apposing metal stent (LAMS) patency, and the need for reinterventions. A total of 216 patients (mean age 64.5 [±13.94] years; 49.1% males) were included. In total, 149 cases (69%) were GOO, 44 (20.4%) cases were bilioenteric anastomotic strictures or lithiasis in altered anatomy, 14 cases (6.5%) were ALS, and 9 patients (4.2%) were for ERCP in altered anatomy after EUS-GG. Overall, EUS-GE was performed in 181 patients (83.8%), EUS-JJ in 44 cases (20.4%), and EUS-GG in 10 (4.6%). Technical success was 94.91%, and clinical success was 93.66%. The adverse event (AE) rate was 11.1%. The reintervention rate was 7.69%. The median follow-up was 85 days. In conclusions, EUS-guided GI anastomoses are technically feasible and safe in both malignant and benign diseases.

## Simple Summary

Patients with gastric outlet obstruction (GOO), afferent limb syndrome (ALS), and patients with altered anatomy due to various gastrointestinal (GI) reconstructions needing endoscopy could benefit from endoscopic ultrasound (EUS)-guided GI anastomoses, such as gastroenterostomy (EUS-GE), gastrogastrostomy (EUS-GG) or jejunojejunostomy (EUS-JJ). This study aimed to evaluate EUS-guided GI anastomoses among diverse clinical scenarios in terms of clinical success, safety, and technical analysis. We confirmed in a series of 216 patients that EUS-guided GI anastomoses are technically feasible and safe in both malignant and benign heterogeneous diseases.

## 1. Introduction

The convergence of endoscopic ultrasonography (EUS) and specifically designed lumen-apposing metal stents (LAMS) has opened new perspectives in the field of gastrointestinal (GI) anastomosis [1,2]. The most common condition requiring EUS-gastroenterostomy (GE) is gastric outlet obstruction (GOO) [3,4], which is more frequently malignant (15–25% of patients with pancreatic cancer develop mGOO) [5,6]. Initially, endoscopic duodenal stenting (ES) or surgical gastrojejunostomy were the alternatives for treating patients with GOO. EUS-GE showed significantly lower rates of adverse events (AEs) compared to ES or surgical gastrojejunostomy (SGJ) [7,8]; as a result, it was developed as a valuable alternative option. This technique transcends conventional boundaries, accommodating altered anatomies resulting from prior surgeries, such as Roux-en-Y reconstructions, Billroth II gastrectomy, and bariatric procedures. EUS-GE bridges not only lumens, but also therapeutic possibilities; so, gastrogastrostomy (EUS-GG) and jejunojejunostomy (EUS-JJ) emerge as therapeutic alternatives [9]. They enable EUS-directed transgastric intervention (EDGI), usually through endoscopic retrograde cholangiopancreatography (ERCP, named EDGE [endoscopic ultrasound-directed transgastric ERCP]) in patients with altered anatomy, thereby circumventing the need for invasive surgical approaches, or allow for the treatment of afferent limb syndrome (ALS) [9,10,11]. In addition, EDGE offers a promising alternative to enteroscopy-assisted or laparoscopy-assisted ERCP for Roux-en-Y gastric bypass (RYGB) patients who need endoscopic access to the biliopancreatic limb. The aim of this large multicenter retrospective study was the evaluation of EUS-guided anastomoses of the gastrointestinal (GI) tract in tertiary referral centers in our country with diverse clinical scenarios; technical success, clinical success, safety, and technical analysis were the endpoints.

## 2. Materials and Methods

### 2.1. Study Design

**Figure 1:**
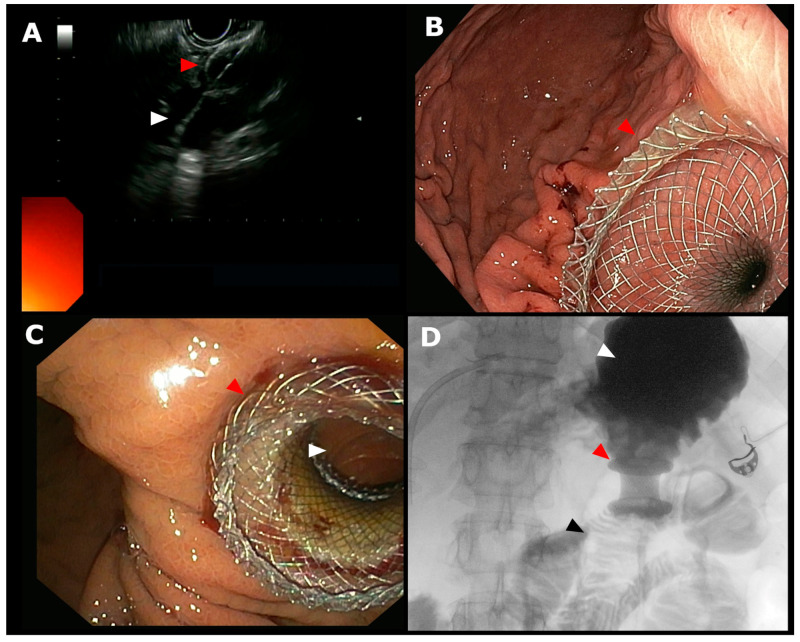
(**A**) Endoscopic ultrasound (EUS) view of lumen-apposing metal stent (LAMS) system deployment with distal tip into the jejunal lumen (white arrow) and distal flange released into the jejunum (red arrow). (**B**) Endoscopic view of proximal flange of LAMS into the gastric cavity when LAMS is not dilated. (**C**) Endoscopic view of a gastrojejunostomy (GJ) after dilation of the LAMS; white arrow indicates jejunal lumen and red arrow indicates proximal flange of the LAMS. (**D**) Radiologic evaluation of the GJ showing contrast dye in the stomach (white arrow) flowing through the LAMS (red arrow) into the jejunum (black arrow).

This is a retrospective multicenter study including all consecutive patients undergoing EUS-guided GI anastomoses (EUS-GE, EUS-GG, and EUS-JJ) with LAMS placement from January 2016 to March 2023 at 10 Italian tertiary centers. Patients were included if they underwent EUS-guided GI anastomoses with LAMS (Figure 1) and suffered from GOO, ALS, or had an altered anatomy (gastric resection, gastric by-pass or other surgical intervention with Roux-en-Y anastomosis) with indication for accessing the bilioenteric anastomotic area.

### 2.2. Population and Outcomes

**Figure 2:**
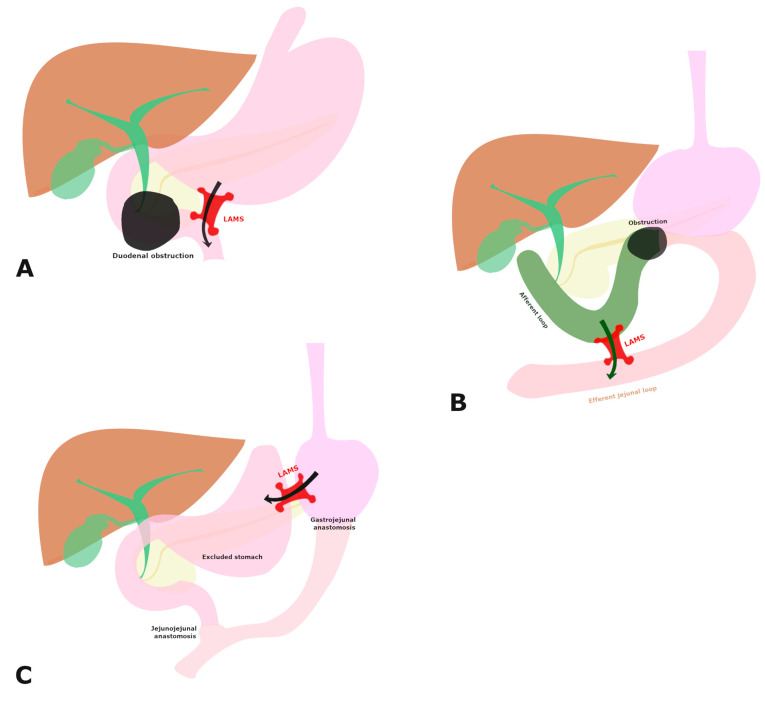
Graphical representation of EUS-guided gastroenterostomy: (**A**) EUS-guided jejunojejunostomy, (**B**), and (**C**) EUS-guided gastrogastrostomy.

The patients were divided into four groups, as follows: group 1 = patients with GOO undergoing EUS-GE; group 2 = patients with ALS undergoing EUS-JJ; group 3 = patients with RYGB needing ERCP and undergoing EUS-guided GI anastomoses; group 4 = patients with altered anatomy due to GI reconstruction undergoing GI-anastomoses for accessing the bilioenteric anastomotic area. The primary outcome was technical success. Secondary outcomes were clinical success, safety, LAMS patency, and the need for reinterventions. Technical and clinical success were defined differently in each group (Supplementary Materials). This study employed three EUS-guided procedures: EUS-GE, EUS-GG, and EUS-JJ (Figure 2). AEs, such as unintentional perforation, bleeding, stent misplacement or migration, and peritonitis, among others, were reported according to the American Society for Gastrointestinal Endoscopy (ASGE) lexicon [12]. In cases of stent misdeployment, we further classified the AE according to a recent misdeployment classification [13].

### 2.3. Statistical Analysis

The data are presented as mean ± standard deviation (SD) and median plus interquartile range (IQR). Comparisons were made by chi-square or Fisher’s exact test for categorical data, and Wilcoxon signed-rank test for non-parametric continuous data. Predictors of technical success, clinical success, AE, and mortality were analyzed by logistic regression analysis. To assess the statistical significance of multiple analyses, the Holm–Bonferroni correction was applied (see Supplementary Materials). Missing data were not included in the analysis. The statistics were processed using SPSS statistical program (version 27.0, SPSS Inc., Chicago, IL, USA) and the statistical software STATA (Statistics and data science, version 18, College Station, TX, USA).

## 3. Results

### 3.1. Characteristics of Population

Ten centers included a total of 216 patients undergoing EUS-guided GI tract anastomoses. The mean age was 64.5 (±13.94) and 49.1% (*n* = 106) were male; 149 (69%) patients had GOO, 14 (6.5%) had ALS, 9 (4.1%) had RYGB and indication for ERCP, and 44 (20.4%) had other altered anatomy and bilioenteric anastomotic strictures. When evaluating the type of procedure, 181 (83.8%) were EUS-GE, 10 (4.6%), were EUS-GG, and 25 (11.6%) were EUS-JJ. The endosonographers mainly used the Hot Axios System (96.7%), and a NAGI stent was used in the remaining cases (3.3%, *n* = 7). Table 1 summarizes the overall characteristics of the patients. Notably, age (*p* < 0.001), ASA score (*p* = 0.016), LAMS diameter (*p* < 0.001), type of LAMS (*p* < 0.001), time of procedure (*p* < 0.001, Figure S1), and postoperative length of stay (LOS) (*p* < 0.001) were statistically different among the four groups (Table S1).

### 3.2. Outcomes

Outcomes based on the type of procedure (EUS-GE, EUS-JJ, and EUS-GG) are shown in Table 2, while those based on the four groups are shown in Table 3. Overall, technical success was 94.91% (CI 95%, 91.11–97.13%), clinical success was 93.66% (CI 95% 89.45–96.26%), and the AE rate was 11.1% (CI 95% 7.58–16.0%) (Table S2). LAMS patency was 97.6% (CI 95% 93.44–98.57%) in a median follow-up of 85 days (IQR, 194 days). Procedure-related mortality included one death, which followed acute abdominal pain due to perforation within seven days (at the second post-procedure day) in a patient with malignant GOO undergoing EUS-GE. Other deaths were due to tumor progression in cases of malignancies, except for one in the benign (bGOO) group, which was due to cardiovascular events (94-year-old woman). In addition, differences in the duration of the procedure (mean difference—MD 14.34 min, *p* = 0.035), and LOS (MD 14.34 days, *p* < 0.001) were related to the AE rate. Furthermore, 33.8% had dilation of the LAMS, showing no differences between the rates of AE (AE with dilation vs. AE without dilation, 12.3% vs. 10.5%; *p* = 0.684). Moreover, when classifying LAMS misdeployment [13], type II was the most common (50%), followed by type I (37.5%) and type IV (12.5%). All cases were managed during the same session. In cases of type I, management included a surgically assisted gastrentero-anastomosis (GEA) and a second successful attempt at EUS-GEA. In type II (*n* = 4), management was always through a second successful EUS-GEA. Type IV (*n* = 1) misdeployment was successfully managed with a same-session duodenal stent placement. Thirteen AEs were reported according to the ASGE lexicon (fatal *n* = 1, severe *n* = 2, moderate *n* = 8, and mild *n* = 2). The relationship between outcomes and clinical/technical features is summarized in Table S3.

### 3.3. Gastric Outlet Obstruction Undergoing EUS-GE

The first group included 149 patients with GOO, 135 (92.5%) of whom had malignant etiology. Mean body mass index at baseline was 21.8 (±4.57), while at the end of follow-up, it was 21.1 (±4.00). Malignancies were due mainly to pancreatic cancer (63%), while the main benign etiologies were GI strictures due to chronic pancreatitis (54.5%, *n* = 6), followed by pyloric stricture after chemotherapy in complete-response patients (18.2%, *n* = 2) (Table 4). Previous attempts to treat GOO were made through endoscopic stricture dilation or duodenal stent placement in 4 (2.8%) and 13 (9%) cases, respectively. Technical success was 94.6%, and the AE rate was 10.1%. The score of GOOSS (Gastric Outlet Obstruction Scoring System [14]) at baseline was predominately zero (53.1%) or one (40.7%), while after proper creation of EUS-GE, the score was predominately two (73.6%) (Figure S2), achieving a clinical success of 94.3%. Furthermore, higher clinical success was associated with a lower ASA score (clinical success when ASA 1 vs. ASA 2 vs. ASA 3 vs. ASA 4: 83.3% vs. 98.1% vs. 93.9% vs. 50%, respectively; *p* = 0.001), and the use of anticoagulant therapy (clinical success when using vs. no use: 84.6% vs. 96.4%; *p* = 0.022 (Table S4). LAMS patency was 98.6% at a median follow-up of 58 days (IQR, 117 days). The reintervention rate in this group was 7.69% (6.1% in malignant etiology vs. 27.3% in benign diseases, *p* = 0.04).

#### 3.3.1. Malignant GOO

In total, 135 patients with mGOO were followed-up for a median time of 57 (IQR 112) days. Technical success was 97%, clinical success was 96.2%, and the AE rate was 9.6%. Six AEs were misdeployment, 50% of which were type II according to a recent misdeployment classification [13]. LAMS for EUS-GE had a diameter of 15 mm in 48.1% of vases, and 20 mm in 51.9%; 27.4% of LAMSs were dilated. LAMS patency was 99.2% during the follow-up period.

#### 3.3.2. Benign GOO (bGOO)

There were 11 patients with bGOO who were followed-up for a median time of 200 (IQR 482) days. Technical success was 90.9%, clinical success was 70%, and the AE rate was 18.2% (two cases, one bleeding within 7 days of the procedure successfully treated conservatively, and one intraprocedural type I misdeployment successfully treated endoscopically with a second attempt at EUS-GE). LAMS for EUS-GE had a diameter of 15 mm in 45.5%, and 20 mm in 54.5%. LAMS removal showed a time to removal of 6 months, with no recurrence of symptoms at 22 months after the procedure. The three reinterventions (27.3%) were one recurrence of symptoms due to stent obstruction after 497 days, treated with the LAMS-in-LAMS technique, and two patients undergoing surgical revision (one of whom after 9 days due to symptom persistence caused by delayed gastric emptying).

### 3.4. Afferent Limb Syndrome Undergoing EUS-JJ

In the second group (*n* = 14), the mean age was 68 (±9.18), and six (42.9%) of the patients were male (Table S5). Only Hot Axios as the type of LAMS was used (100%) in these patients, and the most used diameter was 15 mm (*n* = 11, 78.6%). The duration of the procedure was 38.67 (±16.36) min, and the LAMS was dilated in two cases (14.3%). Technical success was 85.7%, and clinical success was 100%. The procedures did not result in any AE. LAMS patency was 100% at a median follow-up of 69 days (IQR, 100 days).

### 3.5. Patients with RYGB Needing ERCP

The third group included nine patients who had undergone RYGB in the past and needed ERCP. The most common indication was biliary stenosis (44.4%), followed by biliary stones (22.2%). The mean age was 49.3 (±9.53), and two patients (22.2%) were male (Table S6). ERCP was performed in the same session in 66.7% of the cases. Only Hot Axios as the type of LAMS was used (100%) in these patients, and the LAMS diameter was 15 mm in 55.6% (*n* = 6) of patients. The mean duration of the procedure was 85.38 (±49.26) min, and the LAMS was dilated in seven cases (77.8%). Technical success was 100%, and clinical success was 88.9%. The AE rate was 11.1%. The mean LOS was 5.67 (±1.15). LAMS patency was 100% at median follow-up of 315 days (IQR, 310 days).

### 3.6. Patients with GI Reconstruction and Need for Access to Bilioenteric Anastomotic Area

The fourth group included 44 patients with “other anastomoses” needing ERCP for bilioenteric anastomotic strictures. The mean age was 60.7 (±13.17), and 25 patients were male (56.8%) (Table S7). Considering the type of LAMS, Hot Axios was used in 35 patients (83.3%), and the diameter was 15 mm in all cases, while a NAGI stent was used in seven cases (16.7%). The duration of the procedure was 32.92 (±4.5) min, and the LAMS was dilated in 26 cases (59.1%). Technical success was 97.7%, and the AE rate was 18.2%. Initial clinical success was 90.7%, and 87.2% at two weeks. The mean LOS was 2.14 (±1.66). LAMS patency was 93.0% at a median follow-up of 231 days (IQR, 242 days).

## 4. Discussion

EUS-guided GI anastomoses have the potential advantage of reducing the need for surgical interventions across a variety of clinical scenarios [9,15,16]. The current body of robust data supporting this approach is still limited, focusing mainly on EUS-GE for the treatment of GOO. Regrettably, despite dedicated devices (LAMS) facilitating these interventions [17], their use for EUS-guided anastomoses of the GI tract remains off-label, limiting their application to a few tertiary centers [18]. Indeed, 77.7% of endoscopists performed only on-label procedures, and 22.2% performed both on-label and off-label procedures [18]. Therefore, our study presents a comprehensive analysis of 216 patients undergoing EUS-guided anastomoses across ten tertiary centers. Interventions were performed in 69% of patients suffering from GOO, 6.5% with ALS, 4.1% with RYGB needing access to perform ERCP, and 20.4% with various GI reconstructions necessitating access to the bilioenteric anastomotic area. Overall, we found a high technical success rate (94.91%), suggesting an adequate effectiveness of the technique independent of the indication. The need to perform interventional procedures through the LAMS in groups 3 and 4 (EDGI) led to higher rates of LAMS dilation (77.8% and 59.1%, respectively) compared to groups 1 and 2 (EUS-GE for GOO and EUS-JJ for ALS, 25.5% and 14.3%, respectively). Pooled AE with EUS-GE have been reported to be around 12% [19], and our data confirmed this trend, with an AE rate of 11.1%, which was higher when the procedure duration and LOS were longer. The overall AEs were mostly intraprocedural misdeployment (32% of total AE), followed by bleeding (20%), LAMS migration (16%), and stent ingrowth (12%). However, since our population was heterogeneous, we explored the characteristics and outcomes in the four above-mentioned groups.

The first group included the majority of patients (69%) with AEs (10.1%), in line with the literature [7,8]. Recently, prospective comparative studies have found higher rates of clinical and technical success of EUS-GE over ES [20] in patients with mGOO; furthermore, an RCT showed lower frequency of reintervention when performing EUS-GE [21]. We had similar results, with a 94.6% technical success, 94.3% clinical success, and 98.6% LAMS patency in a median follow-up of 58 days. Unfortunately, the retrospective nature of this study led to missing data; so, we must exercise caution in interpreting LAMS patency. In any event, a meta-analysis including patients suffering from both benign and malignant GOO treated with EUS-GE showed high technical (94%) and clinical success (89.9%) [22]. Notably, we included 11 patients with bGOO, which were mostly chronic pancreatitis stricture (54.5%). In the literature, there are scant data available, although a recent study on acute pancreatitis showed a 92.3% rate of technical success and 87.2% of clinical success of EUS-GJ in a median follow-up of 23 months [23]. In our subgroup of bGOO, the median follow-up was 230 days (IQR, 242 days), with a technical success of 90.9%, and a clinical success of 70%. It is clearly fundamental to be highly selective when treating this category of patients. Our data regarding LAMS removal in this subset are scant, so we have data regarding removal for only one patient (after 6 months), showing no recurrence 22 months after the procedure. Considering that LAMS are not designed for indefinite placement, a study on bGOO [23] showed a median time of 9 months for removal (among the 72% of patients with follow-up undergoing LAMS removal).

However, ASA score 4 and the use of anticoagulation drugs were related to lower clinical success among all patients with GOO. These associations could plausibly lead us to consider that patient frailty results in a lower probability of achieving clinical success, perhaps due to the baseline clinical status influencing the capacity of patients to feed themselves even after a technically successful procedure.

In group 2, we included patients with a rare condition (ALS) in which surgery has been the cornerstone of treatment to date [24]. Today, EUS-guided anastomoses are an effective alternative. Our data showed high clinical (100%) and technical success (85.7%). The literature shows higher technical success (95.6%) [25] for ALS, while clinical success (91.1%) is similar to our results. In this group, the 10 mm LAMS was used in four cases (28.6%, Figure S3), two of which had a technical failure, leading to a drop in the rate. Therefore, even if drainage with LAMS through a GI-anastomosis is highly effective, the use of LAMS with a diameter smaller than 15 mm might be less effective, though the few cases in this analysis do not permit recommendation.

Conversely, interventions in group 3 showed high technical success (100%) and clinical success (88.9%). Indeed, the outcomes of EDGE, as a technical success for the creation of gastrogastrostomy/jejunogastrostomy and subsequent ERCP, are reported to be extremely high (99% and 98%, respectively) [10], in agreement with our results. In these cases, clinical success is strictly dependent on the success of ERCP. Overall, we should be careful in correlating clinical outcomes with the EUS-GG performed in these patients. Moreover, since EUS-GI anastomoses in this setting allow for ERCP or other endoscopic interventions during the same session, some authors have reported using devices for LAMS fixation, such as clips or endoscopic sutures [26]. We did not find any fixation in our cohort, though there was a slight propensity to place the 15 mm diameter LAMS (55.6%). The only AE was an intraprocedural misdeployment of a 15 mm LAMS, resolved during the same session with the use of another 15 mm LAMS.

Group 4 encompassed a diverse array of patients, each with various GI reconstructions, necessitating an ERCP, which was performed through the creation of an additional GI anastomosis allowing for ERCP. The primary indication for these procedures was a bilioenteric anastomotic stricture (93.2%), followed by biliary stones (6.8%). Technical success and clinical success in this setting were 97.7% and 90.7%, respectively, though the AE rate was higher in this group (20.5%).

## 5. Conclusions

To conclude, the differences in the follow-up among the groups confirm the heterogeneity of our cohort, proven further by the more frequent presence of malignant patients in groups 1 and 2 compared to the others. Indeed, the results of our study should be interpreted with caution because of several limitations inherent in the above-mentioned heterogeneity, its retrospective design, and the multicenter nature involving different tertiary centers performing non-standard off-label procedures. On the other hand, the strength of this study includes the large number of cases, and the results of outcomes and technical aspects in each group. Finally, our results confirm the high effectiveness and safety of EUS-guided GI-anastomoses in managing different complex gastrointestinal conditions. Our findings highlight the role of patient fragility in achieving clinical success in cases of GOO, suggesting further studies to explore surrogate indexes of the patient’s baseline status [27]. However, further research is needed to optimize patient selection and standardize techniques for minimizing AE and maximizing success.

## Figures and Tables

**Table 1:** Characteristics of the 216 patients.

**Age, Mean ± SD**	64.5 ± 13.94	
**Gender, M, n (%)**	106 (49.1)	
**ASA score, %**	ASA 1	6 (3.6%)
	ASA 2	76 (45.2%)
	ASA 3	81 (48.2%)
	ASA 4	5 (3.0%)
**BMI baseline, mean ± SD (Kg/m^2^)**	22.1 ± 4.81	
**Indications for EUS-guided anastomoses, %**	Gastric outlet obstruction	149 (69%)
	Afferent limb syndrome	14 (6.5%)
	RYGB and indication for ERCP	9 (4.1%)
	GI reconstruction and need to access bilioenteric anastomotic area	44 (20.4%) ▪ 93.2% (*n* = 41) biliary strictures ▪ 6.8% (*n* = 3) lithiasis
**Type of EUS-guided anastomoses, %**	EUS-GE	181 (83.8%)
	EUS-GG	10 (4.6%)
	EUS-JJ	25 (11.6%)
**Type of LAMS**	Hot Axios	207 (96.7%)
	NAGI	7 (3.3%)
**Diameter of LAMS, %**	10 mm	4 (1.9%)
	15 mm	118 (55.1%)
	16 mm	7 (3.3%)
	20 mm	85 (39.7%)
**Dilation of LAMS, %**		73 (33.8%)
**Procedure duration, mean ± SD (min.)**		53.04 ± 25.2

SD: standard deviation; ASA: American Society of Anesthesiologists; BMI: body mass index; EUS: endoscopic ultrasound; RYGB: Roux-en-Y gastric bypass; ERCP: endoscopic retrograde cholangiopancreatography; GI: gastrointestinal; EUS-GE: EUS-guided gastroenterostomy; EUS-GG: EUS-guided gastrogastrostomy; EUS-JJ: EUS-guided jejunojejunostomy; LAMS: lumen-apposing metal stent.

**Table 2:** Outcomes among subgroups based on the type of procedures (EUS-GE, EUS-GG, and EUS-JJ).

	EUS-GE	EUS-GG	EUS-JJ	*p*-Value
**Technical success**	172 (95%)	10 (100%)	23 (92%)	0.613
**Clinical success**	161 (93.6%)	9 (90%)	22 (95.7%)	0.827
**LAMS patency**	167 (97.1%)	10 (100%)	23 (100%)	0.612
**Adverse events**	21 (11.6%)	1 (10%)	3 (12%)	0.986
**Procedure-related mortality** Deaths	1	0	0	-

EUS-GE: EUS-guided gastroenterostomy; EUS-GG: EUS-guided gastrogastrostomy; EUS-JJ: EUS-guided jejunojejunostomy; LAMS: lumen-apposing metal stent.

**Table 3:** Outcomes among the four groups.

	First Group: Patients with GOO Undergoing EUS-GE	Second Group: Patients with ALS Undergoing EUS-JJ	Third Group: Patients with RYGB Needing ERCP Undergoing EUS-Guided GI Anastomoses	Fourth Group: Patients with GI Reconstruction and Need to Access Bilioenteric Anastomotic Area	*p*-Value
**Technical success**	141 (94.6%)	12 (85.7)	9 (100%)	43 (97.7%)	0.298
**Clinical success**	133 (94.3%)	12 (100%)	8 (88.9%)	39 (90.7%)	0.594
**LAMS patency**	139 (98.6%)	12 (100%)	9 (100%)	40 (93%)	0.182
**Adverse events**	15 (10.1%)	0 (0%)	1 (11.1%)	8 (18.2%)	0.135
**Procedure-related mortality** Deaths	1	0	0	0	**-**

GOO: gastric outlet obstruction; EUS-GE: EUS-guided gastroenterostomy; EUS-GG: EUS-guided gastrogastrostomy; EUS-JJ: EUS-guided jejunojejunostomy; LAMS: lumen-apposing metal stent; ERCP: endoscopic retrograde cholangiopancreatography; EUS: endoscopic ultrasound.

**Table 4:** Characteristics of the first group of patients (performing EUS-GE for GOO, *n* = 149).

**Age, Mean ± SD**	66.2 ± 14.05
**Gender, M, n (%)**	73 (49%)
**BMI baseline, mean ± SD (Kg/m^2^)**	21.8 ± 4.57
**BMI at follow-up, mean ± SD (Kg/m^2^)**	21.1 ± 4.00
**Etiology of GOO, n (%)**	
Malignant	135 (92.5%)
Benign	11 (7.5%)
**Malignancies, n (%)**	
Pancreatic cancer	85 (63%)
Duodenal cancer	13 (9.6%)
Metastasis	8 (5.9%)
Cholangiocarcinoma	6 (4.4%)
Ampullary cancer	3 (2.2%)
Other malignancies (lymphoma, retroperitoneal neoplasia, gallbladder cancer, ovarian cancer, neuroendocrine tumor, unknown)	9 (7.3%)
**Benign diseases, n (%)**	
Chronic pancreatitis stricture	6 (54.5%)
Others (pyloric stricture after chemotherapy, anastomotic stricture, peptic stricture, fluid collection after surgery causing compression)	5 (45.5%)
**Site of obstruction**	
Prepyloric/pyloric	20 (13.7%)
Bulb	42 (28.8%)
Second part of duodenum	51 (34.9%)
Third part of duodenum	31 (21.2%)
Jejunum	2 (1.4%)
**Type of LAMS, n (%)**	
Hot Axios	149 (100%)
**Diameter of LAMS, n (%)**	
15 mm	71 (47.7%)
20 mm	78 (52.3%)
**Dilation of LAMS, n (%)**	38 (25.5%)
**Previous stricture dilation, n (%)**	4 (2.8%)
**Previous duodenal stent, n (%)**	13 (9%)
**Procedure duration, mean ± SD (min)**	54.21 ± 22.7
**GOOSS at baseline, %**	
GOOSS 0	77 (53.1%)
GOOSS 1	59 (40.7%)
GOOSS 2	7 (4.8%)
GOOSS 3	2 (1.4%)
**GOOSS after procedure, %**	
GOOSS 0	7 (4.9%)
GOOSS 1	15 (10.4%)
GOOSS 2	106 (73.6%)
GOOSS 3	16 (11.1%)
**Length of post-op stay (days), mean ± SD**	8.33 ± 11.5
**Reintervention rate, %** **Malignant vs. benign, *p*-value** Malignant Benign	7.69% *p* = 0.04 8/132 (6.1%) 3/11 (27.3%)
**Follow-up (days), median (IQR)**	58 (117)

SD: standard deviation; BMI: body mass index; GOO: gastric outlet obstruction; LAMS: lumen-apposing metal stent; GOOSS: Gastric Outlet Obstruction Scoring System.

## Data Availability

Dataset available on request from the authors.

## Supplementary Materials

The following supporting information can be downloaded at: https://www.mdpi.com/article/10.3390/cancers17050910/s1.
