# Correction: Rizzo et al. Endoscopic Ultrasound-Guided Anastomoses of the Gastrointestinal Tract: A Multicentric Experience. *Cancers* 2025, *17*, 910

**DOI:** 10.3390/cancers18020202

**Published:** 2026-01-08

**Authors:** Giacomo Emanuele Maria Rizzo, Chiara Coluccio, Edoardo Forti, Alessandro Fugazza, Cecilia Binda, Giuseppe Vanella, Francesco Maria Di Matteo, Stefano Francesco Crinò, Andrea Lisotti, Marcello Fabio Maida, Giovanni Aragona, Aurelio Mauro, Alessandro Repici, Andrea Anderloni, Carlo Fabbri, Ilaria Tarantino, on behalf of the I-EUS Group

**Affiliations:** 1 Gastroenterology and Endoscopy Unit, Istituto Mediterraneo per i Trapianti e Terapie di alta Specializzazione—IRCCS ISMETT, 90127 Palermo, Italy; itarantino@ismett.edu; 2 Department of Precision Medicine in Medical, Surgical and Critical Care (Me.Pre.C.C.), University of Palermo, 90127 Palermo, Italy; 3 Gastroenterology and Digestive Endoscopy Unit, Forlì-Cesena Hospitals, AUSL Romagna, 47121 Ravenna, Italy; 4 Digestive and Operative Endoscopy Unit, ASST Grande Ospedale Metropolitano Niguarda, 20162 Milan, Italy; 5 Department of Biomedical Sciences, Humanitas University, Via Rita Levi Montalcini 4, Pieve Emanuele, 20090 Milan, Italy; 6 Endoscopy Unit, IRCCS Humanitas Research Hospital, Rozzano, 20089 Milan, Italy; 7 Pancreatobiliary Endoscopy and Endosonography Division, Pancreas Translational and Clinical Research Centre, IRCCS San Raffaele Scientific Institute, 20132 Milan, Italy; 8 Operative Endoscopy Department, Campus Bio-Medico University Hospital, 00128 Rome, Italy; 9 Gastroenterology and Digestive Endoscopy Unit, The Pancreas Institute, 37134 Verona, Italy; 10 Gastroenterology Unit, Hospital of Imola, University of Bologna, 40026 Imola, Italy; 11 Department of Medicine and Surgery, University of Enna ‘Kore’, 94100 Enna, Italy; 12 Gastroenterology and Hepatology Unit, Ospedale Civile, AUSL Piacenza, 29121 Piacenza, Italy; g.aragona@ausl.pc.it; 13 Gastroenterology and Digestive Endoscopy Unit Fondazione I.R.C.C.S. Policlinico San Matteo, 27100 Pavia, Italy; 14 Department of Internal Medicine and Medical Therapeutics, University of Pavia, 27100 Pavia, Italy

## Additional Affiliation

In the published publication [1], there was an error regarding the affiliation for Prof. Alessandro Repici and Dr. Alessandro Fugazza. In addition to affiliation (5) for Prof. Alessandro Repici, the updated affiliation should be included for both Dr. Alessandro Fugazza and Prof. Alessandro Repici: Endoscopy Unit, IRCCS Humanitas Research Hospital, Rozzano, 20089 Milan, Italy. The authors state that the scientific conclusions are unaffected. This correction was approved by the Academic Editor. The original publication has also been updated.
