# Supplementary material for: Endoscopic Ultrasound-Guided Anastomoses of the Gastrointestinal Tract: A Multicentric Experience
Source: Cancers (Basel). 2025 Mar 6;17(5):910. doi: 10.3390/cancers17050910 (PMC11899671; doi:10.3390/cancers17050910)
Supplement: Supplementary file 1 [file cancers-17-00910-s001.zip › cancers-3446610-supplementary.pdf]

SUPPLEMENTARY MATERIALS

Definitions

Technical success was defined as the proper placement of the LAMS between the two intended GI tracts, confirmed by both endoscopic and radiographic evaluation. Moreover, among each of the four groups, technical success was specifically defined as follows: group 1: between stomach and jejunum; group 2: between residual stomach and the excluded gastric cavity; group 3: between afferent and efferent jejunal loop; group 4: between stomach/jejunum and biliary jejunal loop. Clinical success was also defined differently in each group: the improvement of at least 1 point in the Gastric Outlet Obstruction Scoring System (GOOSS) (25) after EUS-GE in the case of GOO (group 1); the resolution of jaundice in the case of EUS-JJ for ALS (group 2) (26); the ability to perform ERCP through the LAMS placed for EUS-GG (group 3); and the ability to perform ERCP through the LAMS placed for creation of EUS-GE or EUS-JJ (group 4). The GOOSS score was defined as follows: 0= no intake, 1= liquid only, 2= soft solids, 3= full diet (25). LAMS patency was defined as the percentage of patients who did not have stent occlusion during the follow-up. Need for re-intervention was defined as the percentage of patients needing interventions for LAMS-related AEs during the follow-up. Procedure time included ERCP or further interventions in case of group 3 and 4.

Statistical analysis

Holm-Bonferroni method: The p-values for all statistical tests were first sorted in ascending order. The adjusted significance threshold for each individual test was then calculated using the formula:

$$\alpha_i = \alpha/n-(i-1)$$

where α is the overall significance level (0.05), n is the total number of tests, and i is the rank of the p-value. Each p-value was compared to its corresponding adjusted threshold. Tests were considered significant (reject) if the p-value was less than or equal to the adjusted threshold.

	pvalue	rank	holm_a~a	reject
1.	.0009	1	.0008475	0
2.	.0009	2	.0008621	0
3.	.0009	3	.0008772	0
4.	.0009	4	.0008929	0
**5.**	**.0009**	**5**	**.0009091**	**1**
**6.**	**.0009**	**6**	**.0009259**	**1**
**7.**	**.0009**	**7**	**.0009434**	**1**
**8.**	**.0009**	**8**	**.0009615**	**1**
**9.**	**.0009**	**9**	**.0009804**	**1**
**10.**	**.0009**	**10**	**.001**	**1**
**11.**	**.0009**	**11**	**.0010204**	**1**
**12.**	**.001**	**12**	**.0010417**	**1**
13.	.004	13	.0010638	0
14.	.016	14	.001087	0
15.	.22	15	.0011111	0
16.	.35	16	.0011364	0
17.	.078	17	.0011628	0
18.	.119	18	.0011905	0
19.	.127	19	.0012195	0
20.	.135	20	.00125	0
21.	.154	21	.0012821	0
22.	.163	22	.0013158	0
23.	.182	23	.0013514	0
24.	.203	24	.0013889	0
25.	.221	25	.0014286	0

26.	.245	26	.0014706	0
27.	.253	27	.0015152	0
28.	.276	28	.0015625	0
29.	.298	29	.0016129	0
30.	.319	30	.0016667	0
31.	.343	31	.0017241	0
32.	.364	32	.0017857	0
33.	.386	33	.0018519	0
34.	.387	34	.0019231	0
35.	.408	35	.002	0
36.	.425	36	.0020833	0
37.	.433	37	.0021739	0
38.	.486	38	.0022727	0
39.	.495	39	.002381	0
40.	.506	40	.0025	0
41.	.564	41	.0026316	0
42.	.571	42	.0027778	0
43.	.584	43	.0029412	0
44.	.594	44	.003125	0
45.	.612	45	.0033333	0
46.	.613	46	.0035714	0
47.	.626	47	.0038462	0
48.	.683	48	.0041667	0
49.	.695	49	.0045455	0
50.	.722	50	.005	0
51.	.812	51	.0055556	0
52.	.827	52	.00625	0
53.	.88	53	.0071429	0
54.	.909	54	.0083333	0
55.	.945	55	.01	0
56.	.953	56	.0125	0
57.	.96	57	.0166667	0
58.	.964	58	.025	0
59.	.986	59	.05	0

SUPPLEMENTARY FIGURES

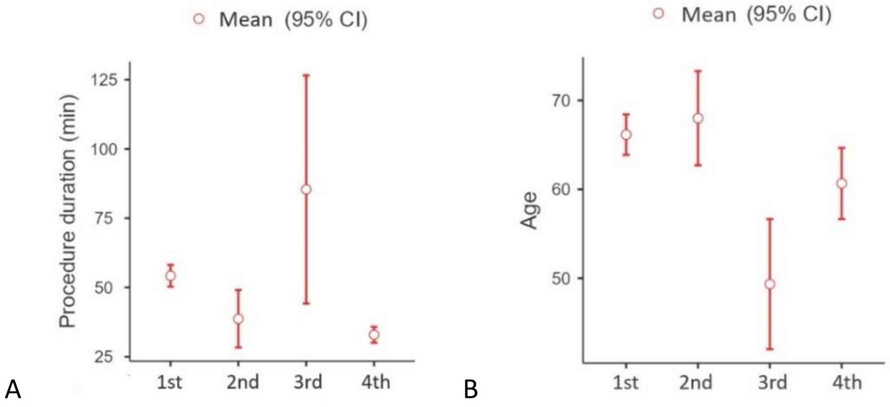

Figure S1. Graphical representation of differences among the four groups in A) mean duration of the procedure and B) mean age.

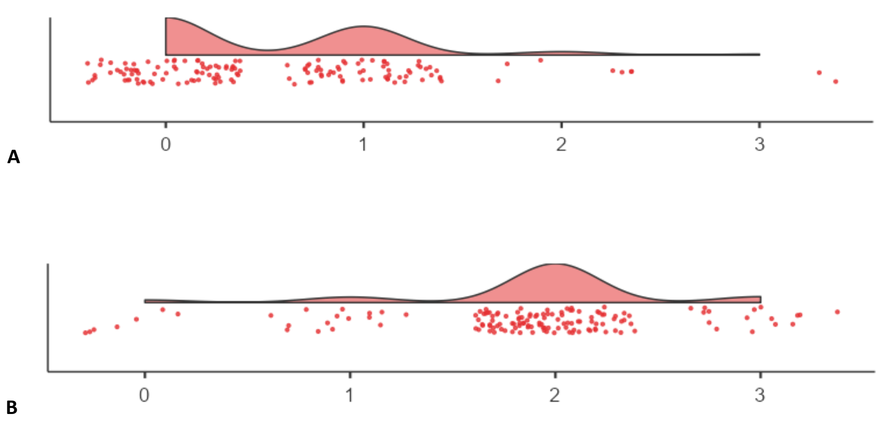

Figure S2. GOOSS (Gastric Outlet Obstruction Scoring System) changes before (A) and after (B) EUS-GE in first group.

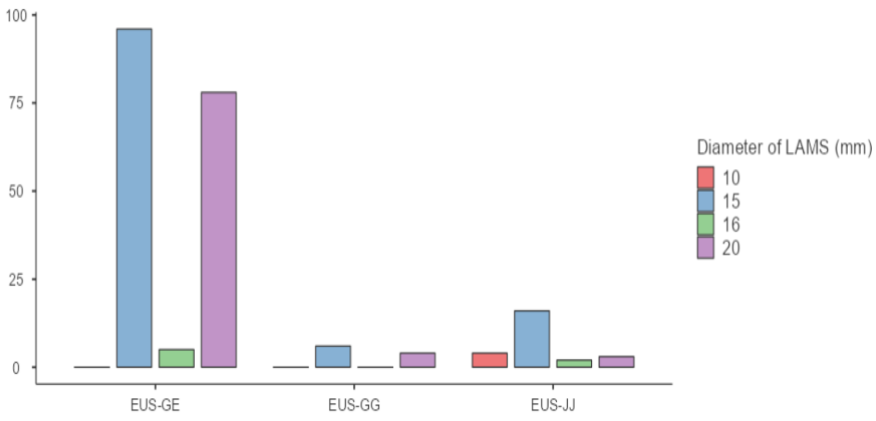

Figure S3. Diameter of LAMS divided for type of procedure.

Table S1. Comparison of the characteristics of patients and outcomes among the four groups.

	First group: patients with GOO undergoing EUS-GE	Second group: patients with ALS undergoing EUS-JJ	Third Group: patients with RYGB needing ERCP undergoing EUS-guided GI anastomoses	Fourth group: patients with GI reconstruction and need to access bilioenteric anastomotic area	p-value
**Age**	66.15 (±14.05)	68.0 (±9.18)	49.33 (±9.53)	60.66 (±13.17)	**P<0.001**
**Gender (M)**	73 (68.9%)	6 (5.7%)	2 (1.9%)	25 (23.6%)	p=0.276
**BMI at baseline**	21.84 (±4.57)	21.68 (±4.11)	28.66 (±7.8)	21.70 (±5.23)	P=0.433
**ASA score**					**p=0.016**
**ASA 1**	6 (100%)	0 (0%)	0 (0%)	0 (0%)	
**ASA 2**	53 (69.7%)	7 (9.2%)	4 (5.3%)	12 (15.8%)	
**ASA 3**	71 (87.7%)	7 (8.6%)	3 (3.7%)	0 (0%)	
**ASA 4**	4 (80%)	0 (0%)	1 (20%)	0 (0%)	
**LAMS diameter**					**P <0.001**
**10 mm**	0 (0%)	4 (100%)	0 (0%)	0 (0%)	
**15 mm**	71 (60.2%)	7 (5.9%)	5 (4.2%)	35 (29.7%)	
**16 mm**	0 (0%)	0 (0%)	0 (0%)	7 (100%)	
**20 mm**	78 (91.8%)	3 (3.5%)	4 (4.7%)	0 (0%)	
**Type of LAMS**					**P<0.001**
**Hot Axios**	149 (100%)	14 (100%)	9 (100%)	35 (83.3%)	
**NAGI**	0 (0%)	0 (0%)	0 (0%)	7 (16.7%)	
**Dilation of the LAMS (yes)**	38 (25.5)	2 (14.3)	7 (77.8%)	26 (59.1%)	**P<0.001**
**Procedure duration (min)**	54.21 (±22.71)	38.67 (±16.36)	85.38 (±49.26)	32.92 (±4.5)	**P<0.001***
**Anticoagulant use**	28 (93.3%)	2 (6.7%)	0 (0%)	0 (0%)	p=0.154
**Length of post-operative stay, (days) mean (SD)**	8.33 (±11.55)	6.85 (±4.91)	5.67 (±1.15)	2.14 (±1.66)	**P<0.001***
**Follow up duration, days median (IQR)**	58 (117)	69 (100)	315 (310)	231 (242)	-
**Overall mortality**	57 (38.5%)	3 (21.3%)	0(%)	1 (2.3%)	**P<0.001***

*significant according to adjusted level of significant after correction with Holm Bonferroni method. GOO: gastric outlet obstruction; ALS: afferent limb syndrome; RYGB: Roux-en-Y gastric bypass; ERCP: endoscopic retrograde cholangiopancreatography

Table S2. Overall outcomes

**Technical success**	205/216	94.91%	CI 95% 91.11-97.13%
**Clinical success**	192/205	93.66%	CI 95% 89.45-96.26%
**LAMS patency**	200/205	97.6%	CI 95% 93.44-98.57%
**Follow-up (days), median**		85 days	IQR 194
**AEs**	24/216	11.1%	CI 95% 7.58-16.0%
**Timing of AEs**			
**Intraoperative**	10/24	41.7%	
**Up to 7 days later**	7/24	29.2%	
**More than 7 days later**	7/24	29.2%	
**Mortality procedure-related**	1/15*	6.67%*	CI 95% 1.19-29.81%

*Available data
LAMS: lumen apposing metal stent; AE: adverse events.

Table S3. Evaluation of the relation between variables and primary outcomes in the entire population (n=216).

	Technical success	p-value	Clinical success	p-value	Safety	p-value
**Age, years**	1.25	0.774	0.91	0.823	2.46	0.408
**Gender male, n (%)**	99 (48.3%)	0.321	94 (49%)	0.464	15 (14.2)	0.245
**BMI at baseline, Kg/m²**	1.67	0.289	0.125	0.944	0.466	0.722
**ASA score,**		0.797		**0.05**		0.506
**ASA 1, n (%)**	6 (100)		**5 (83.3)**		0 (0)	

**ASA 2, n (%)**	73 (96.1)		**68 (93.2)**		5 (6.6)	
**ASA 3, n (%)**	76 (93.8)		**71 (93.4)**		9 (11.1)	
**ASA 4, n (%)**	5 (100)		**3 (60)**		1 (20)	
**LAMS diameter, n (%),**		**<0.001***		0.880		**<0.001***
**10 mm**	**2 (50.0)**		2 (100.0)		**0 (0.0)**	
**15 mm**	**113 (95.8)**		106 (93.8)		**9 (7.6)**	
**16 mm**	**6 (85.7)**		6 (100)		**5 (57.1)**	
**20 mm**	**82 (96.5)**		76 (92.7)		**9 (10.6)**	
**Type of LAMS, n (%)**		0.580		0.514		**<0.001***
**Hot Axios**	196 (95.1)		183 (93.4%)		**18 (8.7)**	
**NAGI**	6 (100)		7 (100)		**4 (66.7)**	
**Dilation of the LAMS, n (%)**	-	-		0.824		0.486
**Dilation**			68 (93.2)		10 (13.7)	
**No dilation**			124 (93.9)		15 (10.5)	
**Procedure duration, min.**	-15.86	0.083	-2.636	0.697	**14.34[§]**	**0.035**
**Anticoagulant use (no use)**	143 (84.1%)	0.244	134 (85.4%)	0.126	3 (10%)	0.909
**Length of post-operative stay, days**	-1.41	0.772	-1.908	0.574	**9.8[§]**	**<0.001***

[§]mean difference. ASA: American Society of Anesthesiologists; BMI: body mass index; LAMS: lumen apposing metal stent
*significant according to adjusted level of significant after correction with Holm Bonferroni method.

Table S4. **A)** Analysis evaluating variables and clinical success among subgroups of patients. **B)** A more detailed overview of the variables with lower p-value.

	**Technical success p-value**				**Clinical success p-value**			
	**First group**	**Second group**	**Third group**	**Fourth group**	**First group**	**Second group**	**Third group**	**Fourth group**
Age	0.812	0.695	-	-	0.964	-	0.564	-
Gender (M)	0.953	0.078	-	-	0.163	-	0.571	-
BMI at baseline	0.960	0.253	-	-	0.945	-	-	-
ASA score	0.495	0.127	-	-	**0.001***	-	0.386	-
LAMS diameter	0.387	0.425	-	-	0.203	-	0.343	-
Procedure duration (min)	0.004	0.584	-	-	0.319	-	0.364	-
Anticoagulant use	0.683	0.119	-	-	0.022	-	-	
Length of post-operative stay (Days)	0.880	0.626	-	-	0.221	-	-	-

ASA: American Society of Anesthesiologists; BMI: body mass index
*significant according to adjusted level of significant after correction with Holm Bonferroni method.

Table S5. Characteristics of second group.

Characteristics of arm 2 (EUS-JJ for afferent limb syndrome), n =14	
**Age, mean ± SD**	68 ± 9.18
**Gender, M, n (%)**	6 (42.9%)
**BMI baseline, mean ± SD (Kg/m^2)**	21.7 ± 4.11
**Type of LAMS, n (%)**	
**Hot Axios**	14 (100%)
**Diameter of LAMS, n (%)**	
**10 mm**	4 (28.6%)
**15 mm**	11 (50.0%)
**20 mm**	3 (21.4%)
**Dilation of LAMS, n (%)**	2 (14.3%)
**Procedure duration, mean ± SD (min.)**	38.67 ± 16.36
**Length of post op stay (days), mean ± SD**	6.85 ± 4.91
**Technical success, %**	85.7 %
**Clinical success, %**	100%
**Adverse events, %**	0%
**LAMS patency, %**	100%
**Follow-up (days), median (IQR)**	69 (100)

SD: standard deviation; BMI: body mass index; LAMS: lumen apposing metal stent

Table S6. Characteristics of third group.

Characteristics of arm 3 (altered anatomy and indication for ERCP), n =9	
**Age, mean ± SD**	49.3 ± 9.53
**Gender, M, n (%)**	2 (22.2%)
**BMI baseline, mean ± SD (Kg/m²)**	28.7 ± 7.8
**Indication for ERCP, n (%)**<br>**Biliary stenosis**<br>**Biliary stones**<br>**Suspicion of ampulloma**<br>**Cholecystitis**<br>**Pancreaticojejunal anastomosis stenosis**	 4 (44.4%)<br>2 (22.2%)<br>1 (11.1%)<br>1 (11.1%)<br>1 (11.1%)
**ERCP at same session, n (%)**	6 (66.7%)
**Type of LAMS, n (%)**	
**Hot Axios**	9 (100%)
**Diameter of LAMS, n (%)**	
**15 mm**	5 (55.6%)
**20 mm**	4 (44.4%)
**Dilation of LAMS, n (%)**	7 (77.8%)
**Procedure duration, mean ± SD (min.)**	85.38 ± 49.26
**Length of post op stay (days), mean ± SD**	5.67 ± 1.15
**Technical success, %**	100 %
**Clinical success, %**	88.9 %
**Adverse events, %**	11.1 %
**LAMS patency, %**	100 %
**Follow-up (days), median (IQR)**	315 (310)

SD: standard deviation; BMI: body mass index; ERCP: endoscopic retrograde cholangiopancreatography; LAMS: lumen apposing metal stent

Table S7. Characteristics of fourth group

Characteristics of arm 4 (other anastomoses for accessing bilioenteric anastomotic area), n =44	
**Age, mean ± SD**	60.7 ± 13.17
**Gender, M, n (%)**	25 (56.8 %)
**BMI baseline, mean ± SD (Kg/m²)***	21.7 ± 5.23
**Indications**<br>**Biliary stenosis**<br>**Lithiasis**	 41 (93.2%)<br>3 (6.8%)
**Type of procedure, n (%)**	
**EUS-GE**	32 (72.7%)
**EUS-GG**	1 (2.3%)
**EUS-JJ**	11 (25%)
**Type of LAMS, n (%)**	
**Hot Axios**	35 (83.3%)
**NAGI**	7 (16.7 %)
**Diameter of LAMS, n (%)**	
**15 mm**	35 (83.3 %)
**16 mm**	7 (16.7 %)
**Dilation of LAMS, n (%)**	26 (59.1 %)
**Procedure duration, mean ± SD (min.)**	32.92 ± 4.5
**Length of post op stay (days), mean ± SD**	2.14 ± 1.66
**Technical success, %**	97.7 %
**Clinical success, %**	90.7 %
**Adverse events, %**	18.2 %
**LAMS patency, %**	93.0 %
**Follow-up (days), median (IQR)**	231 (242)

SD: standard deviation; BMI: body mass index; LAMS: lumen apposing metal stent

*Data were limited
